# Magenta-Blue Electrofluorochromic Device Incorporating Eu(III) Complex, Anthracene Derivative, and Viologen Molecule

**DOI:** 10.3390/ma15155202

**Published:** 2022-07-27

**Authors:** Kazuki Nakamura, Namiko Yanagawa, Norihisa Kobayashi

**Affiliations:** Graduation School of Engineering, Chiba University, 1-33 Yayoi-cho, Inage-ku, Chiba 263-8522, Japan

**Keywords:** electrofluorochromism, electrochromism, europium(III) complex, intermolecular energy transfer

## Abstract

Electrochemical switching of luminescence color between magenta and blue using two types of luminescent materials and electrochromic molecules was demonstrated based on the control of excited energy transfer through an electrochromic reaction. The magenta photoluminescence, due to the integration of red luminescence from the Eu(III) complex and blue fluorescence from the anthracene derivative, was reversibly modulated to a pure-blue luminescence color by an electrochemical redox reaction. Electrofluorochromism is induced by effective excited energy transfer from the Eu(III) complex to the electrochromic molecule under a redox reaction.

## 1. Introduction

Stimuli-responsive photofunctional materials which alter their optical properties in response to external stimuli have been a subject of growing interest. Their photophysical characteristics, such as light-absorption, light-emission, and light-scattering, are able to modulate in response to various triggers such as optical [1,2,3,4,5], thermal [6,7,8,9,10,11], mechanical [12,13,14,15,16], and electrical stimuli. Among them, electrochemical-responsive photofunctional molecules have attracted considerable attention since electrical inputs can be applied rapidly and repeatedly to the responsive systems. Here, we focus on electrochemical-responsive photofunctional molecules and materials which encompass controllable photophysical behavior, such as photo-luminescence and absorption. For example, electrochromic materials change their light absorption property, i.e., color, reversibly through electrochemical redox reactions. Therefore, they can be desirable candidates for key materials in optical-modulating devices such as anti-glare mirrors, smart windows, and display devices [17,18,19,20,21,22,23]. Additionally, modulation of intensity/wavelength of photoluminescence, i.e., electrofluorochromism, is crucial for application of controllable photoluminescence characters in functional devices such as chemical/biochemical sensors, luminescence devices, and imaging devices [24,25,26,27].

We are interested in the electrochemical manipulation of the photoluminescence of lanthanide(III) (Ln(III)) complexes. The Ln(III) complexes exhibit superior luminescence characteristics, such as sharp emission and long lifetimes for luminescence by inner f–f transition, high luminescence quantum efficiencies, and high transparency in the visible light range because of the antenna effect caused by their ligands called “pseudo-Stokes shift” [28,29,30,31]. Owing to these optical properties, Ln(III) complexes have broad application for bioassays/bioimaging, sensors, and display devices [32,33,34,35]. Previously, we reported electrochemical modulation of both absorption and luminescence using Eu(III) complex, a luminescent Ln(III) complex, as a photoluminescent molecule, and electrochromic viologen derivatives [36,37,38,39]. By developing an electrochemical device comprising an electrolyte solution dissolving the Eu(III) complex and viologen derivatives, the modulation of luminescence and coloration was demonstrated by the use of electrochromic reaction of viologen derivatives. The photoluminescence from Eu(III) complexes was effectively manipulated by energy transfer from the excited Eu(III) ions to the colored viologen derivatives.

Regarding the electrochemical modulation of photoluminescence from the Eu(III) complex, Faulkner et al. reported the synthesis and modulation of the electrochemical luminescence of a ferrocene-appended Eu(III) complex [40,41]. The intensity of red luminescence from the Eu(III) ion of the d–f hybrid complex can be electrochemically modulated by changing the redox state of the ferrocene moiety. Recently, Kim and Hasegawa et al. described the control of the red emission from helical Eu(III) complexes through redox reaction of the Eu(III) complex itself [42]. The Eu(III) complex with a helical ligand received a reversible redox reaction, resulting in large modulation of the red emission.

However, most previous reports on electrofluorochromic devices containing Eu(III) complexes were concerned with only a modulation of luminescence intensity, i.e., ON/OFF switching of the red luminescence from Eu(III) complex. To the best of our knowledge, electrofluorochromic devices that achieve luminescence color control using Eu(III) complexes have rarely been reported [43,44]. These systems needed ten to thirty minutes to change their luminescence color, and the resulting luminescence intensity was still insufficient. In this study, we demonstrate the obvious and quick electrochemical control of luminescence color using two types of luminescent materials (Eu(III) complex and organic fluorophore) and an electrochromic viologen molecule.

In our previous research, strong red luminescence of Eu(III) complex can be controlled very efficiently by electrochromic reaction of small organic molecules; the ON/OFF contrast of luminescence modulation was over 3000:1. This quite efficient switchover was achieved thanks to characteristic f–f transitions in Eu(III) ions. Because of the long-lived excited state of ^5^D_0_ level in Eu(III) ions, quenching efficiency of the Eu(III) complex could be high value even by simply mixing the Eu(III) complex and electrochromic molecule in comparison with conventional fluorescent dyes. Moreover, the required application voltage of this electroflurochromic system was less than 1.0 V, enabling reversible modulation of luminescence without significant damage to electrochromic molecules and Eu(III) complex itself.

**Figure 1:**
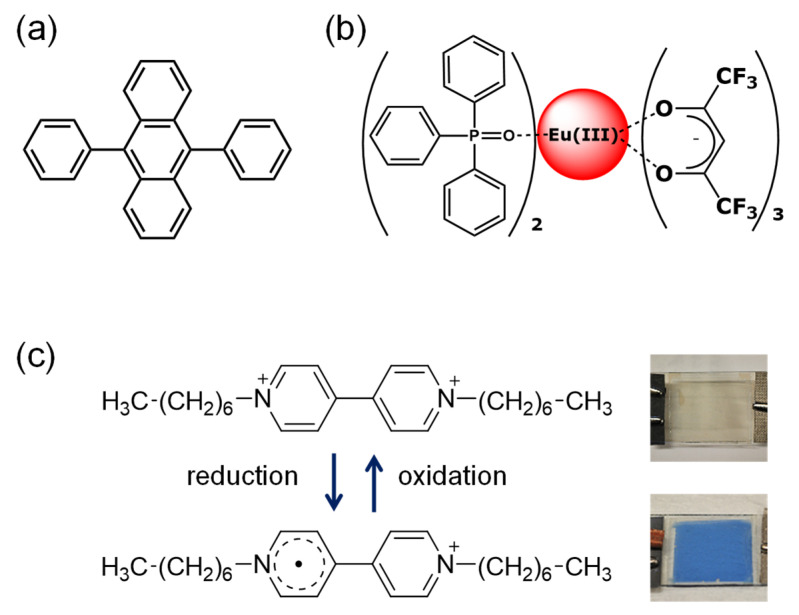
Chemical structure of (**a**) DPA, (**b**) Eu(hfa)_3_(TPPO)_2_ (hfa; hexafluoroacetylacetone), and (**c**) HV^2+^.

From these findings, we focused on a combination of two types of luminophores with quite different lifetimes of their excited states, i.e., Eu(III) complex and fluorescence dye. We employed a red luminescent Eu(III) complex and blue luminescent 9,10-diphenyl anthracene (DPA) as luminescent molecules, and heptyl viologen (HV^2+^) as an electrochromic molecule (chemical structures of these molecules are shown in Figure 1). Using an electrochemical device containing these materials, the magenta photoluminescence due to the integration of red luminescence (phosphorescence) from the Eu(III) complex and blue fluorescence from the organic fluorophore is reversibly modulated to blue luminescence by an electrochemical redox reaction.

## 2. Experimental Section

### 2.1. Materials

Europium(III) acetate n-hydrate (99.9%), hexafluoroacetylacetone (hfa-H_2_), triphenylphosphine oxide (TPPO), the blue fluorescent molecule 9–10 diphenyl anthracene (DPA), and the electrochromic molecule 1,1′-diheptyl-4,4′-bipyridinium dibromide were purchased from Tokyo Chemical Industry Co. (Japan). Solvent consisting of electrolyte solution of propylene carbonate (Kanto Chemical Co., Inc., Tokyo, Japan) was used after removal of water by molecular sieves (Kanto Chemical Co., Inc., Tokyo, Japan). Supporting electrolyte of tetra-n-butylammonium perchlorate (TBAP; Kanto Chemical Co., Inc., Tokyo, Japan) was used without further purification. As transparent electrode, indium tin oxide (ITO; Yasuda, Japan 10 Ω/sq) coated glass electrode was washed by detergent, followed by deionized water twice, and then by acetone using an ultrasonic bath. In addition, UV–O_3_ treatment was carried out before experiments for 20 min to clear away organic surface contamination. The dispersion of ITO (average particle size < 100 nm) in isopropanol (Sigma Aldrich Co., LLC, St. Louis, MO, USA) was used to prepare the ITO nanoparticle-coated electrode.

### 2.2. Synthesis of Red Phosphorescent Eu(III) Complex

Tris(hexafluoroacetylacetonato)europium [Eu(hfa)_3_(H_2_O)_2_]

Eu(hfa)_3_(H_2_O)_2_ was obtained using a literature method [45]. Europium acetate n-hydrate was dissolved in deionized water at room temperature, then, the three-equivalent amount of the liquid hfa-H_2_ was added dropwise. After 3 h stirring, appeared white precipitation was filtered and purified by recrystallization using methanol/water. Yield: 80%. Anal. calcd for C_15_H_7_O_8_F_18_Eu: C, 22.48; H, 0.88%. Found: C, 22.12; H, 1.05%.

Tris(hexafluoroacetylacetonato)europium(III) bis(triphenylphospine oxide) [Eu(hfa)_3_(TPPO)_2_]

Eu(hfa)_3_(TPPO)_2_ was also synthesized using a literature method [45]. Methanol solution containing Eu(hfa)_3_(H_2_O)_2_ (4.3 g, 6 mmol) and TPPO (2.8 g, 10 mmol) was refluxed for 12 h, then the mixture solution was concentrated by evaporation. The pale-yellow crystals of the Eu(hfa)_3_(TPPO)_2_ were obtained by recrystallization of crude compounds from methanol. Anal. calcd for C_51_H_33_O_8_F_18_P_2_Eu: C, 45.96; H, 2.50%. Found: C, 46.20; H, 2.38%.

### 2.3. Preparation of ITO Nanoparticle-Modified Electrodes

ITO particle-modified electrodes were prepared according to a previously reported method [46,47]. The nanosized ITO particle-dispersed isopropanol solution mentioned above was coated by spin-coating method on the conventional flat ITO electrode (500 rpm for 5 s, 1500 rpm for 15 s). Then, these electrodes were heated to 250 °C for 1 h on a heating plate. Average thickness of the prepared ITO particle layer was measured as ca. 1.5 μm using a surface profiler (Surfcorder ET 4000A, Kosaka Laboratory Ltd., Tokyo, Japan).

### 2.4. Fabrication of Electrochemical Cell

The electrolyte solutions for the electrochemical investigations were prepared by dissolving luminescent molecules of Eu(hfa)_3_(TPPO)_2_ (1 mmol L^−1^) and DPA (1 mmol L^−1^), electrochromic molecule of HV^2+^ (5 mmol L^−1^), and supporting electrolyte of TBAP (200 mmol L^−1^) in propylene carbonate. These solutions used for the electrochemical measurements were purged with N_2_ gas for 20 min before each experiment. For comparison, electrolyte solutions containing some molecules were also prepared. A three-electrode electrochemical cell was fabricated using a pre-washed flat ITO glass electrode as the working electrode (active area: 2 cm^2^), Pt wire as the counter electrode, and Ag/AgCl electrode as the reference electrode. A two-electrode electrofluorochromic device was developed using pre-washed flat ITO glass electrodes (active area: 2.25 cm^2^) as the working electrode and an ITO particle-modified electrode as the counter electrode. To develop the two-electrolyte device, a propylene carbonate electrolyte solution was sandwiched between two electrodes, and its electrochemical and photoluminescence properties were evaluated. The inter-electrode distance was maintained at 75 μm using a plastic spacer (Lintec, Tokyo, Japan).

### 2.5. Measurements of Electrochemical Properties

Cyclic voltammetry experiments and chronoamperometry experiments were carried out using a potentio/galvanostat (ALS660A, CH Instruments, Inc., Austin, TX, USA) controlled with a computer. A scan rate in conventional measurement conditions was 50 mV s^−1^. The in situ absorption spectra for the three and two-electrode devices were recorded using a fiber optic spectrometer system (USB2000, Ocean Optics, Orlando, FL, USA) during potential or voltage sweeping. The polarity of the applied voltage in the two-electrode device was defined as positive when the working ITO electrodes were connected to the anode.

In situ electrode potential measurement of the two-electrode device was conducted by associating three potentiostats. The potential measurement needs a voltage source to apply driving voltage between the working and the counter electrodes, and potentiometers to measure each electrode potential of the working and counter electrode vs. the reference electrode. In the present report, a potentiostat (ALS440A, CH Instruments, Inc., Austin, TX, USA) was employed as the voltage source and other two potentiostats (ALS2323, CH Instruments, Inc., Austin, TX, USA and ALS660A) were also placed as potentiometers. To apply voltage in the two-electrode device, the terminal for the working electrode of ALS440A was connected to the flat ITO working electrode, and terminals for the counter and reference electrode were connected to the flat ITO counter electrode or ITO particle-modified electrode. The inter-electrode distance between the working and counter electrodes was set to 10 mm. In addition, the electrochemical cell contained an Ag/AgCl reference electrode between the working and counter electrodes. The potentials of the working and counter electrodes vs. the reference electrode were measured using other potentiostats (ALS2323 and ALS660A) while applying a certain voltage to the two electrodes. During the measurements, absorption changes, CV, and change in electrode potentials of working and counter electrode were simultaneously recorded.

### 2.6. Photophysical Measurements

UV–vis absorption spectra of the two-electrode device (optical path length: 75 μm) were corrected using a spectrophotometer (V-570, JASCO Corporation, Japan). Photoluminescence spectra of the two-electrode device were obtained by a spectrofluorometer (FP-8500, JASCO Corporation, Tokyo, Japan). The excitation wavelength was 333 nm. The measurements of emission lifetimes of the two-electrode devices were performed by fluorescence lifetime spectrometer (Quantaurus-Tau C11367-21, Hamamatsu Photonics K. K., Shizuoka, Japan). The efficiency for the photoluminescence of these electrolyte solutions was measured using an absolute photoluminescence quantum yield spectrometer with integration sphere (Quantaurus-QY C11347-01, Hamamatsu Photonics K. K., Shizuoka, Japan). The solutions used for opto-electrochemical measurements were purged with N_2_ gas for 20 min before each measurement.

## 3. Results and Discussion

### 3.1. Electrochemical Properties of the Mixed Solution Containing Luminescent Materials and Electrochromic Molecules

**Figure 2:**
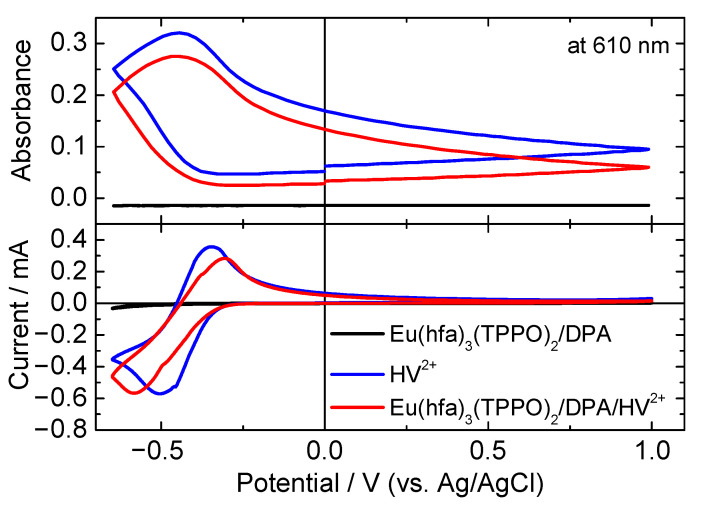
CVs of the electrolyte solutions in three-electrode cell (black line: Eu(hfa)_3_(TPPO)_2_/DPA, blue line: HV^2+^, red line: Eu(hfa)_3_(TPPO)_2_/DPA/HV^2+^).

To clarify the influence of the mixed electrolyte solution on electrochemical behavior, cyclic voltammograms (CVs) and in situ absorbance change at 610 nm were recorded for Eu(hfa)_3_(TPPO)_2_/DPA, HV^2+^, and Eu(hfa)_3_(TPPO)_2_/DPA/HV^2+^ solutions using a three-electrode cell (Figure 2). The electrolyte solution containing Eu(hfa)_3_(TPPO)_2_/DPA didn’t receive an obvious reduction, and oxidation reactions in the measured potential range from +1.0 V to −0.8 V. For the HV^2+^ solution, reductive current and a corresponding oxidative current were observed with peak potentials of −0.50 and −0.35 V, respectively. The absorbance at 610 nm increased as the reductive reaction proceeded. This behavior was attributed to the typical EC reaction of HV^2+^ [48]. As for the Eu(hfa)_3_(TPPO)_2_/DPA/HV^2+^ solution, reductive and oxidative peaks were also recorded at −0.58 and −0.30 V, and the corresponding absorption change was observed to be presumably identical to that of the HV^2+^ solution. With regard to the reversibility of the electron transfer process at the electrode, we were not able to estimate by changing scan rate of the CV measurement because of relatively high resistance of the ITO electrode. The viologen molecule is known to receive highly reversible redox reactions in the 1st reduction process, therefore we expect that the electron transfer process between electrode and viologen molecule in our case would be an intrinsic reversible process.

**Figure 3:**
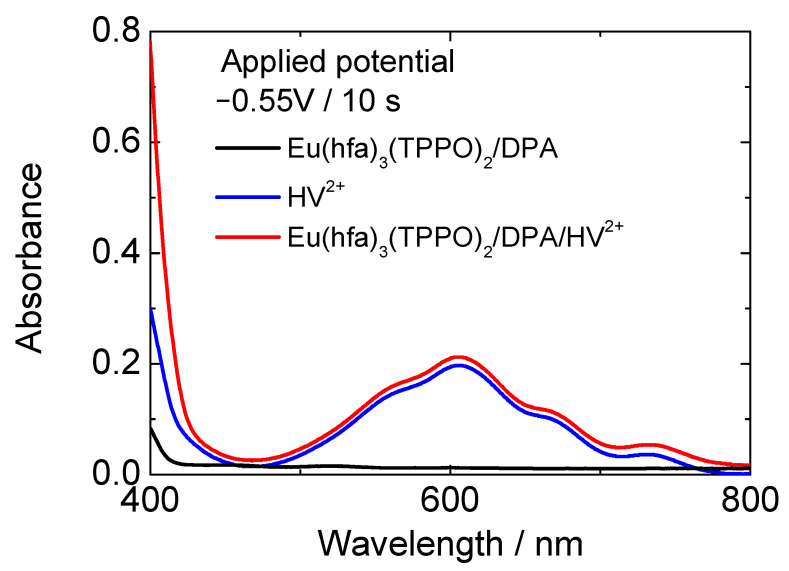
Absorption spectra of the electrolyte solutions in three-electrode cell under application of reduction potential of −0.55 V for 10 s (black line: Eu(hfa)_3_(TPPO)_2_/DPA, blue line: HV^2+^, red line: Eu(hfa)_3_(TPPO)_2_/DPA/HV^2+^).

Figure 3 shows the absorption spectra of the HV^2+^ and Eu(hfa)_3_(TPPO)_2_/DPA/HV^2+^ solutions. When a reduction peak potential of −0.55 V was applied for 10 s, new absorption bands at approximately 400 and 610 nm were observed in both solutions, resulting in change of their color from colorless transparent to cyan color. The coloration of HV^2+^ in the presence of Eu(hfa)_3_(TPPO)_2_ and DPA was consistent with the typical electrochromic behavior of HV^2+^, indicating that Eu(hfa)_3_(TPPO)_2_ and DPA did not have significant influence on the electrochromic behavior of HV^2+^.

**Figure 4:**
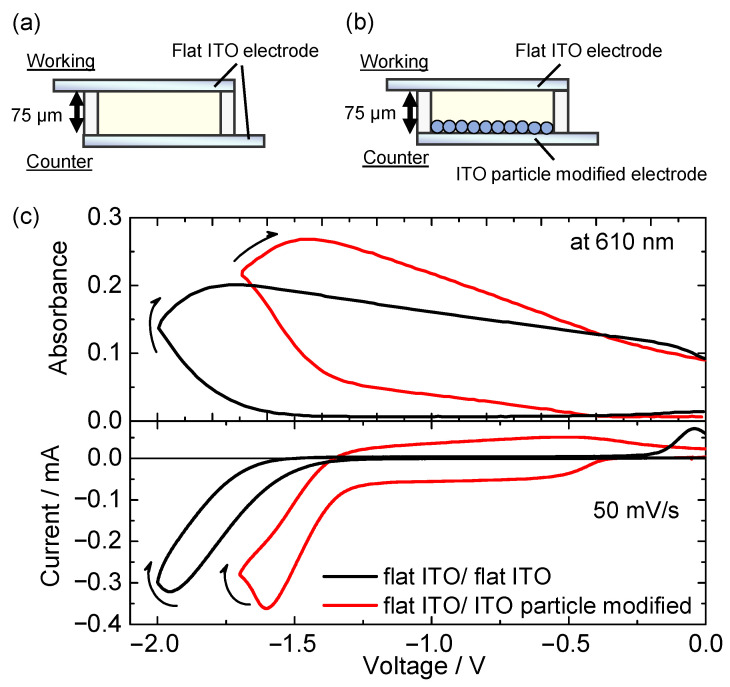
Schematic drawing of the device configuration of (**a**) flat ITO/flat ITO and (**b**) flat ITO/ITO particle-modified electrode. (**c**) CVs of two-electrode electrochemical device (**bottom**) and in situ absorption change at 610 nm (**top**). The electrolyte solution contains Eu(hfa)_3_(TPPO)_2_, DPA, HV^2+^, and supporting electrolyte. (Black line: two flat ITO electrodes, red line: flat ITO electrode and ITO particle-modified electrode).

We then fabricated two-electrode electrochemical devices to investigate the luminescence control of the Eu(hfa)_3_(TPPO)_2_/DPA/HV^2+^ solution by an electrochemical redox reaction. Figure 4a,b shows the schematic of the electrochemical devices, which consist of two flat ITO electrodes (Figure 4a, flat ITO/flat ITO), or a flat ITO electrode as the working side and an ITO particle-modified electrode as the counter side (Figure 4b, flat ITO/ITO particle-modified electrode). A propylene carbonate solution dissolving Eu(hfa)_3_(TPPO)_2_, DPA, HV^2+^, and supporting electrolyte was inserted between the two electrodes with inter-electrode distance of 75 μm. For solution-based electrochromic devices, the maximum absorbance (color intensity) of the device is affected by the distance because colored molecules generated on the working electrode diffuse into the solution and they are deactivated by collision with the counter electrode (or redox species from the counter electrode). However, color intensity can also be controlled by waveform and magnitude of application voltage. In the case of the electrofluorochromic device in this study, since the required coloration of the viologen molecule for luminescence control was quite low (~0.01), wide electrode distance and large amount of reaction charge were not necessary.

Figure 4c describes the CVs and in situ change of absorbance at 610 nm of the two-electrode device. With regard to the two-electrode device using flat ITO electrode as the counter electrode (Figure 4a and black line in Figure 4c), with voltage sweeping to the negative direction from 0 V, the redox current ascribable to electrochemical reduction of HV^2+^ on the working electrode was detected from −1.4 V. According to the reduction of the HV^2+^ to form HV^·+^, the absorbance of reduced HV^2+^ (i.e., HV^·+^) at around 610 nm increased from −1.4 V. In contrast, in the two-electrode device with the ITO particle-modified electrode as a counter side (Figure 4band red line in Figure 4c), the redox current was observed from a lower voltage of −0.5 V than that of the flat ITO/flat ITO device. As the redox current flowed, the absorbance of the HV^2+^ molecules increased. Thus, by introducing the ITO particle-modified electrode as the counter side, a significant reduction in the driving voltage of the electrochromic device was achieved.

To investigate the difference in the driving voltage between the two types of devices, we measured the electrode potentials of both electrodes of working side and counter side relative to the Ag/AgCl reference electrode during application of certain voltages between the working and counter electrodes. These experiments involve one voltage source for applying a voltage between the working and counter electrodes in the two-electrode device, and two potentiometers for measurement of the electrode potentials of the working or counter side [49,50].

**Figure 5:**
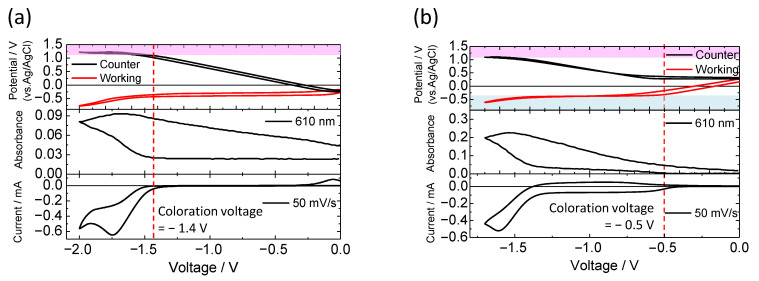
CVs of two-electrode electrochemical device (bottom), in situ absorption change at 610 nm (middle), and in situ electrode potentials (vs. Ag/AgCl) of working and counter electrodes. (**a**) Flat ITO/flat ITO device and (**b**) flat ITO/ITO particle-modified electrode device.

The changes in the electrode potentials of the working and counter sides were monitored with the change in the applied voltage to the devices. Figure 5a shows the CV of the flat ITO/flat ITO device (bottom) and the in situ change in electrode potentials relative to the Ag/AgCl reference electrode (top). As discussed above, the redox current began to flow between the working and counter electrodes above application voltage of −1.4 V. When voltage of −1.4 V was applied between the two electrodes, the electrode potential of the working electrode was −0.3 V (vs. Ag/AgCl), which corresponds to the reduction potential of HV^2+^, whereas the potential of the counter electrode reached +1.1 V (vs. Ag/AgCl). This potential of +1.1 V (vs. Ag/AgCl) was attributed to the anodic reaction of the DPA molecule [51,52]. These behaviors indicated that the redox charge consumed by the reduction of HV^2+^ molecule on the working electrode was compensated by the oxidation of the DPA molecule on the counter electrode.

Concerning to the electrofluorochromic device with flat ITO electrode/ITO particle-modified electrode (Figure 5b), the redox reaction started from a lower voltage of −0.5 V; in this device, the electrode potential of the working electrode swiftly reached the reduction potential of HV^2+^ (−0.3 V vs. Ag/AgCl) in comparison with the flat ITO/flat ITO device. For the device involving the ITO particle-modified electrode as the counter side, the electrode potential of the counter side was almost unchanged before the start of the redox reaction because of the large capacitance of the ITO particle layer. This electrochemical response indicates that the ITO particle-modified electrode effectively compensated for the equivalent amount of charge consumed by the reduction of HV^2+^ on the working electrode by charging the electrical double layer (without the electrochemical reaction of the DPA). Above −1.4 V, the electrode potential of the counter electrode reached the oxidation potential of the DPA molecule (+1.1 V vs. Ag/AgCl), leading to a large increase in the redox current and absorbance of the reduced HV^2+^ molecule. These results suggest that the charge accumulation due to the electrical double layer of the ITO particle-modified electrode effectively reduced the driving voltage of the two-electrode device, preventing the electrochemical oxidation of the DPA molecule on the counter electrode.

### 3.2. Electrochemical Control of Luminescence Color

**Figure 6:**
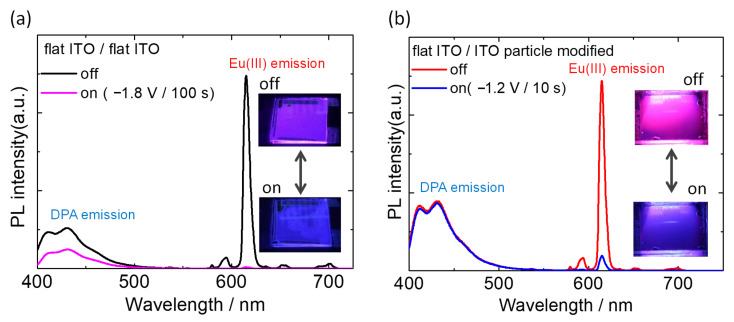
PL spectra of the electrofluorochromic devices containing Eu(hfa)_3_(TPPO)_2_/DPA/HV^2+^ solution with or without application of reduction voltage. (**a**) The device consists of two flat ITO electrodes, and (**b**) flat ITO electrode and ITO particle-modified electrode. The luminescent molecules in the electrolyte solution were photo excited by 333 nm light.

The redox behavior of the electrofluorochromic devices was elucidated; therefore, we demonstrated the electrochemical change of luminescence color using the two devices. Photoluminescence spectra of the two-electrode devices were recorded with and without the application of a redox voltage (Figure 6). Excited light (333 nm) was irradiated from the working electrode side, and the emitted light from the device was detected on the same side.

In the case of the flat ITO/flat ITO device (Figure 6a), two types of broad blue fluorescence luminescence bands were observed from the DPA molecule at approximately 430 nm, and sharp red emissions due to the f–f transition of Eu(hfa)_3_(TPPO)_2_ were observed prior to voltage application. The sharp emission bands at 590, 615, 650, and 700 nm were ascribed to ^5^D_0_ → ^7^F_J_ (J = 1, 2, 3, and 4) transitions. As an additive mixture of luminescence colors, the device showed magenta luminescence, as shown in Figure 6a. By applying a reduction voltage of HV^2+^ on the working side (−1.8 V), the intensity of the red emission from the Eu(III) complex was almost quenched within a few tens of seconds. Consequently, the luminescence color from the device turned blue because the blue fluorescence from the DPA molecule could be observed under the application of voltage. This quenching of Eu(III) luminescence was induced by efficient energy transfer from the photoexcited Eu(III) complex to the reduced HV^2+^ species, as previously reported. However, as the redox reaction of the device proceeded, the blue fluorescence from DPA was also reduced by half, and the reversibility of the luminescence modulation was significantly low. These limitations of the device are mainly due to the undesired but unavoidable oxidative reaction of the DPA molecule on the counter electrode, as shown in Figure 5a.

In contrast, a flat ITO/ITO particle-modified device can be operated at a lower voltage than the DPA molecule, which does not undergo redox reactions, as discussed in the previous section. Therefore, a bias voltage of −1.2 V, which was lower than the redox voltage of DPA, was applied to the electrofluorochromic device to modulate the photoluminescence (Figure 6b). Prior to the voltage application, magenta photoluminescence was observed as the same as the flat ITO/flat ITO device. By applying a redox voltage of −1.2 V, the cathodic reaction of HV^2+^ was initiated, and the color of the device slightly turned to cyan. Within 10 s of the voltage application, the red emission from Eu(hfa)_3_(TPPO)_2_ was reduced by 94%, whereas the blue fluorescence of DPA was almost unchanged.

**Figure 7:**
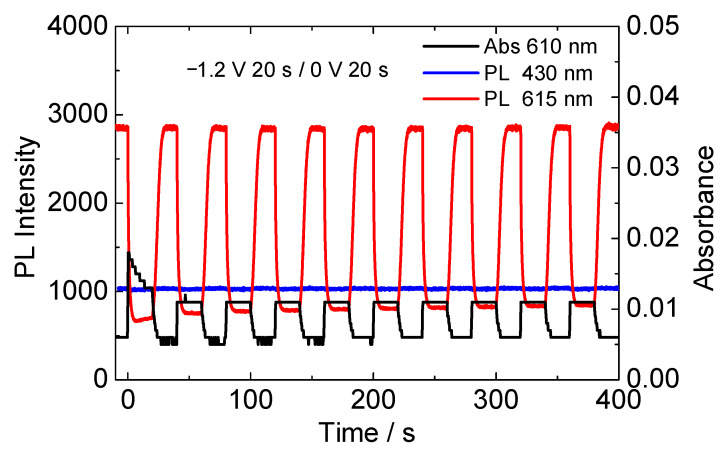
Repeated change in absorption (610 nm), and PL intensities of DPA (430 nm) and Eu(hfa)_3_(TPPO)_2_ emissions (615 nm) of the electrofluorochromic device consisting of flat ITO electrode and ITO particle-modified electrode. Successive voltage for coloration and bleaching of the HV^2+^ were applied to the device (−1.2 V/20 s and 0 V/20 s) repeatedly. Excitation wavelength was 333 nm.

Figure 7 shows the change in absorption of reduced HV^2+^ (610 nm), and PL intensities of DPA (430 nm) and Eu(hfa)_3_(TPPO)_2_ emissions (615 nm) of the electrofluorochromic device with ITO particle-modified counter electrode during repeated application of coloration and bleaching voltage (−1.2 V/20 s and 0 V/20 s). By applying the coloration voltage, the absorbance of the reduced HV^2+^ at 610 nm increased slightly, and this electrochromic reaction was highly reversible. With an increase in the 610 nm absorption, the red luminescence from Eu(hfa)_3_(TPPO)_2_ at 615 nm immediately deceased, and the PL intensity recovered to its initial value with the bleaching reaction of HV^2+^. While the red luminescence of the Eu(III) complex was continually modulated by the voltage application, the blue fluorescence from the DPA molecule at 430 nm remained constant in intensity, as shown by the blue line in Figure 7. Owing to the difference in the response of the luminescence intensity to the bias voltage, a reversible electrofluorochromic reaction between magenta and blue was successfully achieved. At its present state, relatively stable electrochemical switching of luminescence color has been confirmed in several tens of cycles. However, since the device was not encapsulated to prevent atmospheric and moisture contamination yet, the device characteristics such as electrochromic reversibility and uniformity of electrochromic reaction gradually depredated after several tens of cycles. Essentially, the electrochromic molecule of viologen used in this research is well known to repeat stable electrochromic reactions under appropriate conditions. Therefore, by properly sealing the device or conducting performance tests in a glovebox, these electofluorochromic reactions are expected to be repeated more stably.

### 3.3. Photophysical Measurements of the EFC Device

**Figure 8:**
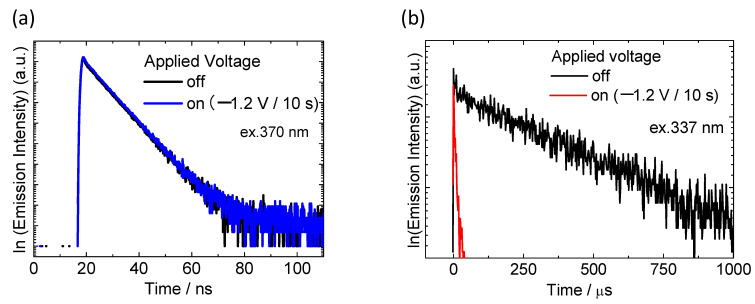
Emission decay curves of (**a**) DPA and (**b**) Eu(hfa)_3_(TPPO)_2_ in the electrofluorochromic device with and without application of coloration voltage (−1.2 V/10 s). Excitation wavelength were 370 nm for DPA and 337 nm for Eu(hfa)_3_(TPPO)_2_.

To discuss the mechanism of luminescence color modulation, the photophysical properties of the electrofluorochromic device with an ITO particle-modified counter electrode were investigated in detail. The measurement of luminescence decay curves of DPA and Eu(hfa)_3_(TPPO)_2_ in the device were performed before and after the application of the coloration voltage (Figure 8). In the case of DPA fluorescence (Figure 8a), the lifetime of the excited state of DPA before voltage application was calculated to be ca. 7 ns. The fluorescence lifetime of DPA showed almost no change when the coloration voltage was applied; this result agreed with the luminescence spectra shown in Figure 6b. In contrast, Eu(hfa)_3_(TPPO)_2_ exhibited a completely different behavior from that of DPA. The luminescence lifetime of Eu(hfa)_3_(TPPO)_2_ in the electrofluorochromic device was 530 μs, which is significantly longer than that of DPA fluorescence owing to the long-lived f–f transition of the Eu(III) ion. The long lifetime of Eu(hfa)_3_(TPPO)_2_ was abruptly shortened to ca. 30 μs by the voltage application (Figure 8b, red line).

The efficiency for energy transfer from photo-excited luminescence materials of DPA or Eu(hfa)_3_(TPPO)_2_ to colored HV^·+^ was calculated using Equation (1):

(1) $$\eta_{\mathrm{ENT}}=1-\frac{\tau_{\mathrm{ON}}}{\tau_{0}}$$

where *η*_ENT_, *τ*_0_, and *τ*_ON_ are the energy transfer efficiency from the luminescent molecule to HV^·+^, the emission lifetime of DPA or Eu(hfa)_3_(TPPO)_2_ without voltage application, and the emission lifetime of the DPA or Eu(hfa)_3_(TPPO)_2_ under voltage application, respectively [36]. Table 1 lists the photophysical parameters of DPA and Eu(hfa)_3_(TPPO)_2_ in the electrofluorochromic device. The energy transfer efficiency from excited DPA molecules to colored HV^·+^ was significantly low (0.003), whereas that from excited Eu(hfa)_3_(TPPO)_2_ to colored HV^·+^ reached 0.94. The large difference in the energy transfer efficiencies of DPA and Eu(hfa)_3_(TPPO)_2_ facilitates a significant change in the photoluminescence color of the electrochemical device. In addition, we calculated the radiative rate (*k*_r_), nonradiative rate (*k*_nr_), and energy transfer rate (*k*_ENT_) using Equations (2) and (3):

(2) $$k_{r}=\frac{\phi_{0}}{\tau_{0}}$$

(3) $$k_{\mathrm{ENT}}=\frac{\eta_{\mathrm{ENT}}}{\tau_{0}\left(1-\eta_{\mathrm{ENT}}\right)}$$

where *Φ*_0_ is the luminescence quantum yield of luminescent molecules in the device without voltage application [36]. By comparing the values of *k*_r_ and *k*_ENT_, *k*_r_ was sufficiently larger than *k*_ENT_ in the case of the DPA molecules because of the short-lived S_1_–S_0_ fluorescence of DPA. Therefore, the fluorescence intensity of DPA was unaffected by the electrochromism of HV^2+^. For Eu(hfa)_3_(TPPO)_2_, the *k*_ENT_ value was 46 times larger than *k*_r_, resulting in efficient quenching of the red luminescence of Eu(hfa)_3_(TPPO)_2_. A large difference in the *k*_r_ value between the fluorescent DPA and f–f transition of Eu(hfa)_3_(TPPO)_2_ affects the dependence of the luminescence intensity on HV^2+^ electrochromism.

## 4. Conclusions

In this study, electrochemical switching of luminescence color between magenta and blue was successfully demonstrated using an electrochemical device containing a red luminescent Eu(III) complex, blue luminescent DPA, and an electrochromic viologen molecule. The magenta photoluminescence, due to the integration of red luminescence from the Eu(III) complex and blue fluorescence from the DPA molecule, was reversibly modulated to blue DPA luminescence by an electrochemical redox reaction. This brilliant electrofluorochromism was induced by effective excited energy transfer from Eu(hfa)_3_(TPPO)_2_ to the electrochromic viologen molecule. We presume that Eu(III) complex-based electrofluorochromic devices will contribute to the development of novel photofunctional devices such as chemical/biological sensor systems, security systems, and display devices.

## Figures and Tables

**Table 1:** Photophysical parameters of the DPA and Eu(hfa)_3_(TPPO)_2_ in the electrofluorochromic device.

	*τ*_0_ (s)	*τ*_ON_ (s)	*η*_ENT_	*Φ*_0_	*k*_r_ (s^−1^)	*k*_nr_ (s^−1^)	*k*_ENT_ (s^−1^)
DPA	7.03 × 10^−9^	7.01 × 10^−9^	0.003	0.77	1.1 × 10^8^	3.3 × 10^7^	4.1 × 10^5^
Eu(hfa)_3_(TPPO)_2_	5.30 × 10^−4^	0.30 × 10^−4^	0.94	0.34	6.7 × 10^2^	1.3 × 10^3^	3.1 × 10^4^

## Data Availability

The data presented in this study are available on reasonable request from the corresponding author. The data are not publicly available as the data forms part of an ongoing study.

## References

[1] Matsuda K., Irie M. (2004). Diarylethene as a Photoswitching Unit. J. Photochem. Photobiol. C Photochem. Rev..

[2] de Silva A.P., McClenaghan N.D. (2004). Molecular-Scale Logic Gates. Chem.-A Eur. J..

[3] Granqvist C.G., Lansåker P.C., Mlyuka N.R., Niklasson G.A., Avendaño E. (2009). Progress in Chromogenics: New Results for Electrochromic and Thermochromic Materials and Devices. Sol. Energy Mater. Sol. Cells.

[4] Kawai T., Sasaki T., Irie M. (2001). A Photoresponsive Laser Dye Containing Photochromic Dithienylethene Units. Chem. Commun..

[5] Müller-Buschbaum K., Beuerle F., Feldmann C. (2015). MOF Based Luminescence Tuning and Chemical/Physical Sensing. Microporous Mesoporous Mater..

[6] Granqvist C.G., Green S., Niklasson G.A., Mlyuka N.R., von Kræmer S., Georén P. (2010). Advances in Chromogenic Materials and Devices. Thin Solid Films.

[7] Lin J., Lai M., Dou L., Kley C.S., Chen H., Peng F., Sun J., Lu D., Hawks S.A., Xie C. (2018). Thermochromic Halide Perovskite Solar Cells. Nat. Mater..

[8] Ohtani S., Gon M., Tanaka K., Chujo Y. (2017). A Flexible, Fused, Azomethine–Boron Complex: Thermochromic Luminescence and Thermosalient Behavior in Structural Transitions between Crystalline Polymorphs. Chem.-A Eur. J..

[9] Smith C.R., Sabatino D.R., Praisner T.J. (2001). Temperature Sensing with Thermochromic Liquid Crystals. Exp. Fluids.

[10] Tashiro K., Ono K., Minagawa Y., Kobayashi M., Kawai T., Yoshino K. (1991). Structure and Thermochromic Solid-state Phase Transition of Poly (3-alkylthiophene). J. Polym. Sci. Part B Polym. Phys..

[11] Yoshida M., Sääsk V., Saito D., Yoshimura N., Takayama J., Hiura S., Murayama A., Põhako-Esko K., Kobayashi A., Kato M. (2022). Thermo-and Mechano-Triggered Luminescence ON/OFF Switching by Supercooled Liquid/Crystal Transition of Platinum(II) Complex Thin Films. Adv. Opt. Mater..

[12] Kato M., Ito H., Hasegawa M., Ishii K. (2019). Soft Crystals: Flexible Response Systems with High Structural Order. Chem.-A Eur. J..

[13] Ito H., Saito T., Oshima N., Kitamura N., Ishizaka S., Hinatsu Y., Wakeshima M., Kato M., Tsuge K., Sawamura M. (2008). Reversible Mechanochromic Luminescence of [(C_6_F_5_Au)_2_(μ-1,4-Diisocyanobenzene)]. J. Am. Chem. Soc..

[14] Yagai S., Seki T., Aonuma H., Kawaguchi K., Karatsu T., Okura T., Sakon A., Uekusa H., Ito H. (2016). Mechanochromic Luminescence Based on Crystal-to-Crystal Transformation Mediated by a Transient Amorphous State. Chem. Mater..

[15] Sagara Y., Kubo K., Nakamura T., Tamaoki N., Weder C. (2017). Temperature-Dependent Mechanochromic Behavior of Mechanoresponsive Luminescent Compounds. Chem. Mater..

[16] Yoshida M., Kato M. (2018). Regulation of Metal–Metal Interactions and Chromic Phenomena of Multi-Decker Platinum Complexes Having π-Systems. Coord. Chem. Rev..

[17] Tsuboi A., Nakamura K., Kobayashi N. (2014). Multicolor Electrochromism Showing Three Primary Color States (Cyan-Magenta-Yellow) Based on Size- and Shape-Controlled Silver Nanoparticles. Chem. Mater..

[18] Cummins D., Boschloo G., Ryan M., Corr D., Nagaraja Rao S., Fitzmaurice D. (2000). Ultrafast Electrochromic Windows Based on Redox-Chromophore Modified Nanostructured Semiconducting and Conducting Films. J. Phys. Chem. B.

[19] Mortimer R.J., Dyer A.L., Reynolds J.R. (2006). Electrochromic Organic and Polymeric Materials for Display Applications. Displays.

[20] Somani P.R., Radhakrishnan S. (2003). Electrochromic Materials and Devices: Present and Future. Mater. Chem. Phys..

[21] Mondal S., Chandra Santra D., Ninomiya Y., Yoshida T., Higuchi M. (2020). Dual-Redox System of Metallo-Supramolecular Polymers for Visible-to-Near-IR Modulable Electrochromism and Durable Device Fabrication. ACS Appl. Mater. Interfaces.

[22] Kobayashi N., Watanabe Y., Ibata Y., Nakamura K. Reflective and Emissive Dual Mode Display Cell with Electrochromism and Electrochemiluminescence. Proceedings of the International Conference on Digital Printing Technologies 2013.

[23] Watanabe Y., Suemori K., Hoshino S. (2016). Electrochromic Response Characteristics of Dye-Modified Porous Electrodes Affected by the Porous Film Structure. Chem. Lett..

[24] Miomandre F., Audebert P., Miomandre F., Audebert P. (2017). Luminescence in Electrochemistry.

[25] Sun J., Liang Z. (2016). Swift Electrofluorochromism of Donor-Acceptor Conjugated Polytriphenylamines. ACS Appl. Mater. Interfaces.

[26] Audebert P., Miomandre F. (2013). Electrofluorochromism: From Molecular Systems to Set-up and Display. Chem. Sci..

[27] Suzuki T., Sato T., Zhang J., Kanao M., Higuchi M., Maki H. (2016). Electrochemically Switchable Photoluminescence of an Anionic Dye in a Cationic Metallo-Supramolecular Polymer. J. Mater. Chem. C.

[28] Bünzli J.C.G., Piguet C. (2005). Taking Advantage of Luminescent Lanthanide Ions. Chem. Soc. Rev..

[29] Eliseeva S.V., Bünzli J.C.G. (2010). Lanthanide Luminescence for Functional Materials and Bio-Sciences. Chem. Soc. Rev..

[30] Wang C.-M., Pan M.-F., Chen Y.-C., Lin H.-M., Chung M.-Y., Wen Y.-S., Lii K.-H. (2017). Two Polymorphs of an Organic−Zincophosphate Incorporating a Terephthalate Bridging Ligand in an Unusual Bonding Mode. Inorg. Chem..

[31] Wang C.-M., Pan M.-F., Lin Y.-J., Chung M.-Y., Wen Y.-S., Chang Y., Lin H.-M., Hsu T. (2018). A Series of Organic–Inorganic Hybrid Zinc Phosphites Containing Extra-Large Channels. Inorg. Chem..

[32] Tsukube H., Shinoda S. (2002). Lanthanide Complexes in Molecular Recognition and Chirality Sensing of Biological Substrates. Chem. Rev..

[33] Kuriki K., Koike Y., Okamoto Y. (2002). Plastic Optical Fiber Lasers and Amplifiers Containing Lanthanide Complexes. Chem. Rev..

[34] Hirai Y., Nakanishi T., Miyata K., Fushimi K., Hasegawa Y. (2014). Thermo-Sensitive Luminescent Materials Composed of Tb(III) and Eu(III) Complexes. Mater. Lett..

[35] Hasegawa M., Ohmagari H. (2020). Helicate Lanthanide Complexes: The Luminescent Elements. Chem. Lett..

[36] Kanazawa K., Komiya Y., Nakamura K., Kobayashi N. (2017). Red Luminescence Control of Eu(iii) Complexes by Utilizing the Multi-Colored Electrochromism of Viologen Derivatives. Phys. Chem. Chem. Phys..

[37] Kanazawa K., Nakamura K., Kobayashi N. (2015). Electrochemical Luminescence Modulation in a Eu(III) Complex-Modified TiO_2_ Electrode. J. Mater. Chem. C.

[38] Nakamura K., Kanazawa K., Kobayashi N. (2011). Electrochemically Controllable Emission and Coloration by Using Europium(III) Complex and Viologen Derivatives. Chem. Commun..

[39] Kanazawa K., Nakamura K., Kobayashi N. (2018). A Viologen-Eu(III)-Modified TiO_2_ Electrode for Electroswitchable Luminescence and Coloration. ChemistrySelect.

[40] Tropiano M., Kilah N.L., Morten M., Rahman H., Davis J.J., Beer P.D., Faulkner S. (2011). Reversible Luminescence Switching of a Redox-Active Ferrocene-Europium Dyad. J. Am. Chem. Soc..

[41] Lehr J., Tropiano M., Beer P.D., Faulkner S., Davis J.J. (2015). Reversible Redox Modulation of a Lanthanide Emissive Molecular Film. Chem. Commun..

[42] Kim Y., Ohmagari H., Saso A., Tamaoki N., Hasegawa M. (2020). Electrofluorochromic Device Based on a Redox-Active Europium(III) Complex. ACS Appl. Mater. Interfaces.

[43] Mukaigawa M., Ohno H. (1998). Electrochemical Switching of Fluorescence Emission between Red and Blue from Complexed Europium Ions in Poly(Ethylene Oxide). J. Electroanal. Chem..

[44] Mukaigawa M., Ohno H. (1998). Control of Fluorescence Emission Color and Intensity by Electrochemical Redox Reaction of Complexed Europium Ions in PEO. Solid State Ionics.

[45] Nakamura K., Hasegawa Y., Kawai H., Yasuda N., Kanehisa N., Kai Y., Nagamura T., Yanagida S., Wada Y. (2007). Enhanced Lasing Properties of Dissymmetric Eu(III) Complex with Bidentate Phosphine Ligands. J. Phys. Chem. A.

[46] Liang Z., Yukikawa M., Nakamura K., Kobayashi N. (2018). A Novel Organic Electrochromic Device with Hybrid Capacitor Architecture towards Multicolour Representation. Phys. Chem. Chem. Phys..

[47] Araki S., Nakamura K., Kobayashi K., Tsuboi A., Kobayashi N. (2012). Electrochemical Optical-Modulation Device with Reversible Transformation between Transparent, Mirror, and Black. Adv. Mater..

[48] Mortimer R.J. (1999). Organic Electrochromic Materials. Electrochim. Acta.

[49] Tsuneyasu S., Watanabe Y., Nakamura K., Kobayashi N. (2017). In Situ Measurements of Electrode Potentials of Anode and Cathode in Organic Electrochromic Devices. Sol. Energy Mater. Sol. Cells.

[50] Liang Z., Nakamura K., Kobayashi N. (2019). A Multicolor Electrochromic Device Having Hybrid Capacitor Architecture with a Porous Carbon Electrode. Sol. Energy Mater. Sol. Cells.

[51] Minami H., Ichikawa T., Nakamura K., Kobayashi N. (2019). Electrochemically Triggered Upconverted Luminescence for Light-Emitting Devices. Chem. Commun..

[52] Tsuneyasu S., Ichikawa T., Nakamura K., Kobayashi N. (2017). Electrochemical Stability of Diphenylanthracene and Its Effect on Alternating-Current-Driven Blue-Light Electrochemiluminescence Properties. ChemElectroChem.

